# Ten-Year Reintervention-Free Survival After Surgical Aortic Valve Replacement for Different Prosthetic Aortic Valve Models: Comparison Using Real-World Data From the Netherlands Heart Registration

**DOI:** 10.1093/icvts/ivag240

**Published:** 2026-08-31

**Authors:** Hector W L de Beaufort, Maaike M Roefs, Edgar J Daeter, Wilson W L Li, Wim Jan P van Boven, Niels P van der Kaaij, Dennis van Veghel, Cardiothoracic Surgery Registration Committee of the Netherlands Heart Registration, S Bramer, R A F de Lind van Wijngaarden, B M J A Koene, J A Bekkers, G J F Hoohenkerk, A L P Markou, A de Weger, P Segers, D Stecher, R G H Speekenbrink, V G Hindori, W W L Li, E J Daeter, M M Mokhles, I J Wijdh – den Hamer

**Affiliations:** Department of Cardiothoracic Surgery, St. Antonius Hospital, 3435 CM Nieuwegein, The Netherlands; Netherlands Heart Registration, 3511 EP Utrecht, The Netherlands; Department of Cardiothoracic Surgery, St. Antonius Hospital, 3435 CM Nieuwegein, The Netherlands; Department of Cardiothoracic Surgery, Radboud University Medical Center, 6525 GA Nijmegen, The Netherlands; Department of Cardiothoracic Surgery, Amsterdam University Medical Center,1105 AZ Amsterdam, The Netherlands; Department of Cardiothoracic Surgery, Erasmus Medical Center, 3000 CA Rotterdam, The Netherlands; Netherlands Heart Registration, 3511 EP Utrecht, The Netherlands

**Keywords:** aortic valve replacement, long-term outcomes, registry

## Abstract

**Objectives:**

To compare long-term outcomes across different models of mechanical and biological aortic valve prostheses after surgical aortic valve replacement (SAVR) in the Netherlands.

**Methods:**

Data from the Netherlands Heart Registration (NHR) on isolated, primary SAVR procedures performed from 2007 to 2018 in adult patients were used, excluding active endocarditis. Ten-year survival and freedom from valve-related reintervention were estimated for models that were implanted >200 times, using Cox proportional hazards models for survival and Fine and Gray’s models with death as the competing event for freedom of reintervention.

**Results:**

For 11 139 patients treated with a biological prosthetic aortic valve, 10-year survival was highest for the Perimount Magna Ease (72.6%) and Epic (70.0%), and significantly lower for the Perimount Magna (65.1%), Trifecta (63.1%), Perimount (61.4%), Freedom Solo (51.5%), and Mitroflow (50.7%). Ten-year freedom from reintervention was highest for the Perimount Magna (99.3%), and significantly lower for the Perimount Magna Ease (98.2%), Perimount (98.2%), Epic (97.3%), Trifecta (96.8%), Freedom Solo (93.2%), and Mitroflow (90.5%). For 2123 patients treated with a mechanical prosthetic aortic valve, 10-year survival was highest for the Regent (91.2%) and not significantly different for the Open Pivot (89.2%), Slimline (87.5%), HP (87.3%), and significantly lower for the Carbomedics Reduced (87.0%). Ten-year freedom from reintervention was highest for the Carbomedics Reduced (97.1%), not significantly different for the Open Pivot (96.1%), Slimline (96.5%), Regent (95.9%), and significantly lower for the HP (94.9%).

**Conclusions:**

Differences in 10-year outcomes after SAVR were observed for the different prosthetic aortic valve models that were used in the NHR, especially for biological prosthetic aortic valve models, with survival varying from 50.7% to 72.6% and freedom from reintervention from 90.5% to 99.3%.

## Graphical Abstract

**Figure:**
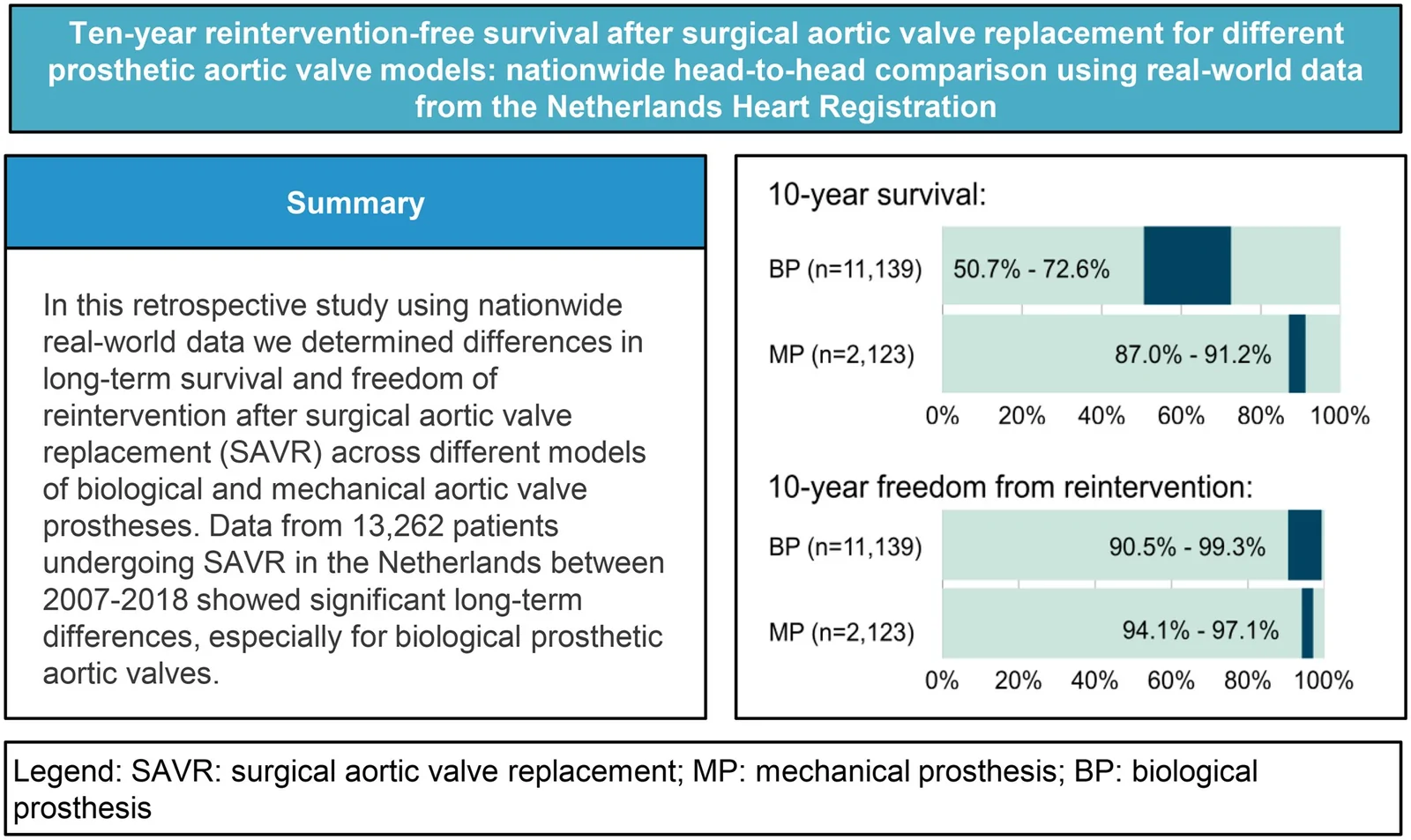

## INTRODUCTION

Surgical aortic valve replacement (SAVR) relies on the implantation of either a mechanical (MP) or a biological (BP) prosthetic aortic valve. Use of an MP is generally favoured in patients under 60 years old, while a BP is reserved for older patients, due to a reduced durability of BP compared to MP.^1^ Less is known about differences in long-term outcomes between different models of MP and BP. Currently used MP models rely on 2 carbon leaflets rotating over a fixed range with pivots on the inside of the valve ring.^2^ Differences between MP models, which all have good reported durability,^3–7^ depend on purity of the carbon, design of the pivot, or location of the leaflets and pivots. The design of currently available BP is more variable, with use of either porcine valve tissue or bovine pericardial tissue, which is mounted on a stent frame or not, and with different tissue preservation techniques. Different BP thus vary in haemodynamic performance and failure mechanisms.^8^ Most available literature on long-term outcomes of different models of prosthetic aortic valves are single-centre experiences, and most involve a single valve model.^9–13^

There are few studies comparing the outcomes across different models. Utilizing comprehensive national databases, such as the Netherlands Heart Registration (NHR), offers the opportunity to conduct analyses of prosthetic aortic valve models over extended periods. Previous registry-based studies from the United Kingdom and from Sweden have underscored variations in outcomes, such as mortality and reintervention rates, among different models of mechanical and biological aortic valve prostheses.^14–16^ Building on this body of knowledge, the hypothesis of this study is that in a more recent, nationwide cohort in which concomitant coronary surgery was excluded, there is a relation between outcomes and the implanted prosthetic aortic valve model. The aim was to compare long-term outcomes, specifically mortality and risk of reintervention, of different models of aortic valve prostheses using real-world data of all isolated SAVR procedures performed in the Netherlands in the 12-year period between 2007 and 2018.

## PATIENTS AND METHODS

### Study population

The methods used for data collection and the definition of end-points used for this study were published previously.^17^ To summarize briefly, the NHR is a nationwide registry containing data of all cardiac surgical interventions in the Netherlands from 2007, with regular checks on the quality of data collection.^18^^,^^19^ For this study, isolated, primary SAVR procedures that were performed between January 2007 and December 2018 were included. Exclusion criteria were: unknown implanted valve type, active endocarditis, prior cardiac surgery, or SAVR combined with another surgical intervention.

All analyses were performed on fully anonymized data. This study complies with the Declaration of Helsinki, and the data use was approved by the Medical Research Ethics Committees United (reference No. W19.270), which issued a waiver for informed consent for the current analysis of anonymized data.

### Endpoints

Mortality during follow-up was noted, with the mortality status of included patients checked on 1 May 2020 or later depending on the timing of data retrieval, using the national Personal Records Database. Follow-up was administratively censored at a maximum of 10 years after the initial SAVR procedure. Reinterventions were defined as any intervention on the aortic valve after the initial SAVR procedure, including open surgical and transcatheter interventions, regardless of the underlying cause or indication, including endocarditis, structural valve degeneration, thrombosis, and paravalvular leak. The occurrence of a reintervention was checked using the NHR database and the unique patient identifier within this database; the first aortic valve reintervention after the primary isolated SAVR was used. Ten-year survival and 10-year freedom from reintervention were co-primary outcomes.

### Baseline characteristics

Baseline characteristics that were analysed were preoperative patient characteristics, including age, body mass index (BMI), body surface area (BSA) and logistic EuroSCORE I, and specifications of the prosthetic aortic valve implanted in the procedure, including valve prosthesis manufacturer, model and size.

### Statistical analysis

Categorical variables were expressed as numbers and percentages and continuous variables as mean with standard deviation (SD) or median with interquartile range (IQR) after evaluation of normality, which was performed using visual inspection of histograms and q-q plots and the Kolmogorov-Smirnov test. Differences in categorical variables were assessed using the chi-squared test and differences in non-normally distributed continuous variables using the Kruskal-Wallis test. To ensure adequate sample sizes for multivariable Cox regression, long-term outcomes were assessed only for valve types implanted more than 200 times during the study period. Biological and mechanical prostheses were analysed separately.

Ten-year survival was assessed using the Cox proportional hazards model that included age, BSA, logistic EuroSCORE I, and valve size (ranging from 19 to 29) as independent factors, stratified by type of prosthesis. The predicted cumulative survival was plotted in survival curves including 95% confidence intervals (CIs).

The risk of reintervention within 10 years after SAVR was analysed using the Fine and Gray’s model in which mortality was accounted for as a competing risk for reintervention and in which age, BSA, logistic EuroSCORE I, and valve size (ranging from 19 to 29) were used as independent factors, stratified by type of prosthesis. The predicted freedom from reintervention of the Fine and Gray model was plotted in a survival curve.

Outcomes of the multivariable regression models were presented as hazard ratios (HRs) with 95% CIs. The proportional hazard assumption was evaluated using Schoenfeld residuals. Time-dependence of covariate effects was evaluated in all models using global and covariate-specific tests. *P*-values of <.05 were considered statistically significant. To analyse differences across prosthetic valve models, the model with the highest adjusted 10-year survival or freedom from reintervention was used as the reference. In case the outcome, duration of follow-up or one of the factors (age, BSA, EuroSCORE I, or valve size) was missing, the intervention was excluded from the survival analysis.

Sensitivity analyses were performed to assess potential residual confounding by prosthesis–patient mismatch (PPM). A proxy variable was defined as prosthesis size indexed to BSA and categorized into quartiles. This variable was added to the multivariable models for both survival and reintervention, as described above. For survival analyses, model comparison was performed using likelihood ratio testing. For reintervention analyses, robustness was assessed by evaluating changes in effect estimates.

Analyses were performed using SPSS Statistics for Windows, Version 26.0 (IBM) and R (version 4.2.1).

## RESULTS

Between 2007 and 2018, 16 956 patients underwent a primary isolated SAVR with a known valve type, of which 13 355 (78.8%) received a BP and 3601 (21.2%) a MP aortic valve. **Table S2** shows all implanted BP and MP models with their manufacturer information during the study period. **Table 1** shows the baseline characteristics of the 11 139 BP and 2123 MP valves that were included in the long-term analyses presented in this study (see **Figure 1**). Baseline characteristics differed significantly between valve types for both BP and MP (*P* < 0.001, **Table 1**).

**Figure 1.**
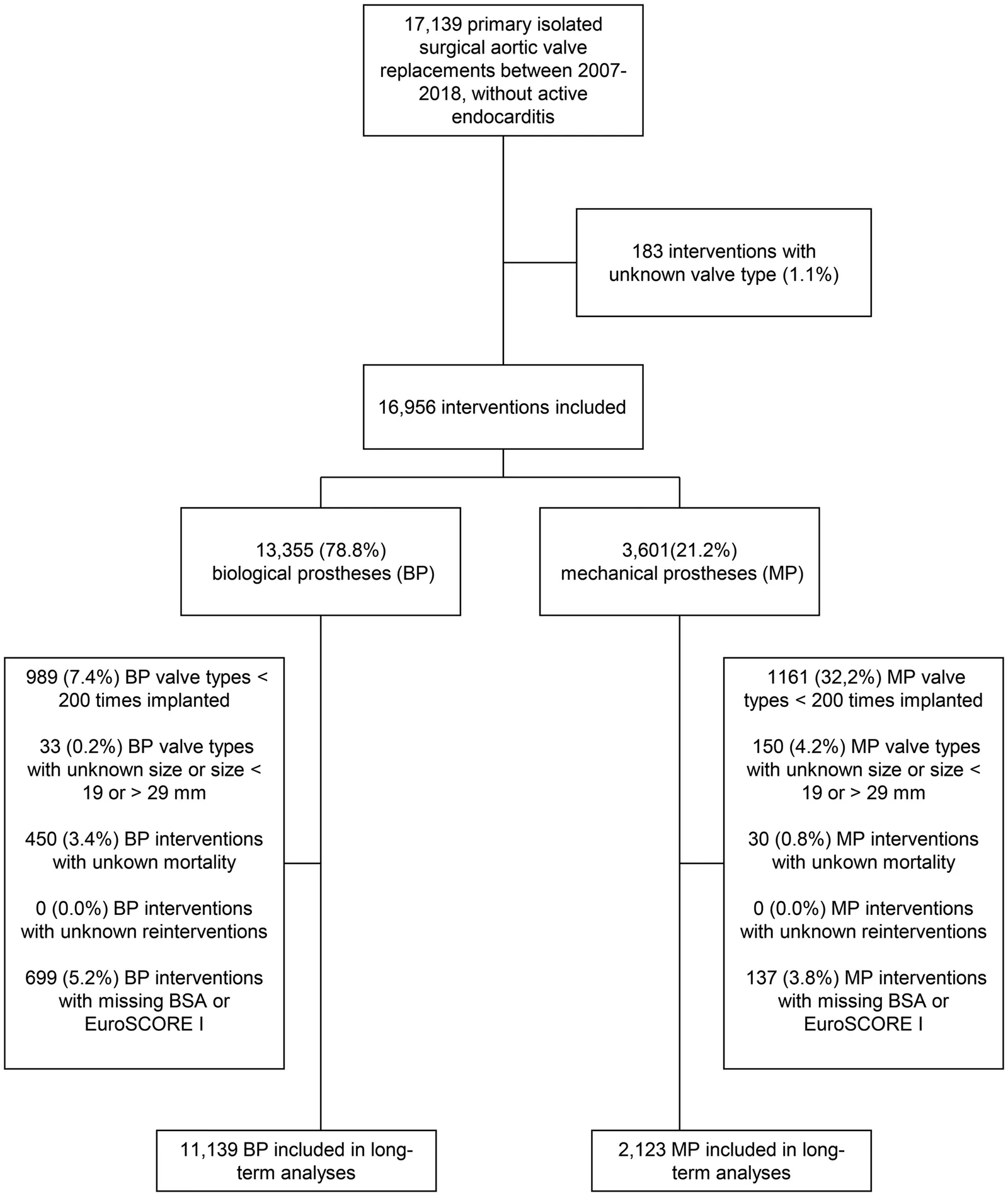
Flowchart Representing Inclusion and Exclusion of Interventions. Abbreviations: BP, biological prostheses; MP, mechanical prostheses

**Table 1.** Baseline Characteristics of Prosthetic Aortic Valves by Model of Biological and Mechanical Prostheses Included in Long-Term Analyses

			Age (years)	Sex (male)	BSA (m^2^)^#^	EuroSCORE I	Mortality in follow-up	Reintervention in follow-up
			Median [Q1, Q3]	*n* (**%**)	Median [Q1, Q3]	Median [Q1, Q3]	*n* (**%**)	*n* (**%**)
**Manufacturer**	**Biological prosthesis**							
Edwards	Perimount Magna Ease	*n* = 4149	71 [66, 77]	2251 (54.3)	1.95 [1.82, 2.09]	4.95 [3.24, 7.47]	706 (17.0)	76 (1.8)
Edwards	Perimount	*n* = 2436	73 [68, 78]	1479 (60.7)	1.94 [1.80, 2.09]	5.43 [3.44, 8.19]	679 (27.9)	44 (1.8)
Edwards	Perimount Magna	*n* = 697	73 [67, 78]	354 (50.8)	1.90 [1.77, 2.06]	5.31 [3.49, 7.87]	225 (32.3)	7 (1.0)
Edwards	Intuity Elite	*n* = 311	73 [68, 77]	147 (47.3)	1.92 [1.78, 2.06]	5.10 [3.33, 6.99]	29 (9.3)	9 (2.9)
Sorin	Freedom Solo	*n* = 550	75 [70, 79]	198 (38.1)	1.90 [1.75, 2.02]	6.17 [4.38, 8.91]	210 (40.4)	29 (5.6)
Sorin	Mitroflow	*n* = 1199	74 [69, 78]	531 (44.3)	1.90 [1.77, 2.05]	6.29 [4.28, 8.87]	467 (38.9)	93 (7.8)
Corcym	Perceval	*n* = 278	75 [70, 79]	118 (42.4)	1.94 [1.80, 2.08]	6.15 [4.36, 8.30]	50 (18.0)	5 (1.8)
St Jude	Epic	*n* = 575	73 [68, 78]	340 (59.1)	1.94 [1.80, 2.07]	5.40 [3.39, 8.16]	140 (24.3)	20 (3.5)
St Jude	Trifecta	*n* = 974	74 [68, 79]	390 (40.0)	1.90 [1.75, 2.04]	5.86 [3.76, 8.23]	201 (20.6)	36 (3.7)
	*P*-value		*P < .001*	*P < .001*	*P < .001*	*P <.001*		
	**Overall**	***n* = 11 139**	**73 [67, 78]**	**5808 (52.1)**	**1.93 [1.79, 2.07]**	**5.39 [3.50, 7.98]**	**2707 (24.3)**	**319 (2.9)**
**Manufacturer**	**Mechanical prosthesis **							
St Jude	Regent	*n *= 417	56 [50, 61]	238 (57.1)	1.98 [1.80, 2.13]	2.08 [1.51, 3.37]	40 (9.6)	21 (5.0)
Corcym	Carbomedics—Reduced	*n *= 771	56 [50, 61]	547 (70.9)	2.04 [1.89, 2.17]	2.08 [1.51, 2.86]	81 (10.5)	23 (3.0)
Medtronic	Open pivot	*n* = 300	59 [53, 65]	204 (68.0)	2.00 [1.87, 2.10]	2.27 [1.51, 3.67]	38 (12.7)	13 (4.3)
Corcym	Slimline	*n* = 393	58 [52, 63]	245 (62.3)	1.99 [1.82, 2.14]	2.26 [1.63, 3.30]	27 (6.9)	14 (3.6)
St Jude	HP	*n* = 242	57 [51, 61]	178 (73.6)	2.02 [1.90, 2.14]	2.08 [1.51, 2.85]	28 (11.6)	15 (6.2)
	*P*-value		*P < .001*	*P < .001*	*P < .001*	*P < .001*		
	**Overall**	***n *= 2123**	**57 [51, 62]**	**1412 (66.5)**	**2.01 [1.86, 2.15]**	**2.08 [1.51, 3.13]**	**214 (10.1)**	**86 (4.1)**

# BSA: body surface area

in bold values for all types of biological and mechanical prosthesis combined

### Biological prosthetic aortic valves

During the study period, BPs were implanted in 13 355 patients. The Perimount Magna Ease was the most frequently implanted BP (31.7%; *n* = 4228), followed by the Perimount (23.2%; *n* = 3102), Mitroflow/Crown (10.8%; *n* = 1441), and Trifecta (8.3%; *n* = 1106) prostheses (**Table S12**). Median age for each model of BP is shown in **Figure 2A**. The Inspiris, Freestyle, and Avalus prostheses were implanted in significantly younger patients and/or with a significantly lower logistic EuroSCORE I than patients treated with other models of BP. The distribution of BP size, ranging from 19 to 29 mm, is shown in **Figure S1**, where size S of the Perceval prosthesis was considered 21 mm, size M was 23 mm, size L was 25 mm, and size XL was 27 mm, based on information provided in the product catalogue.

**Figure 2.**
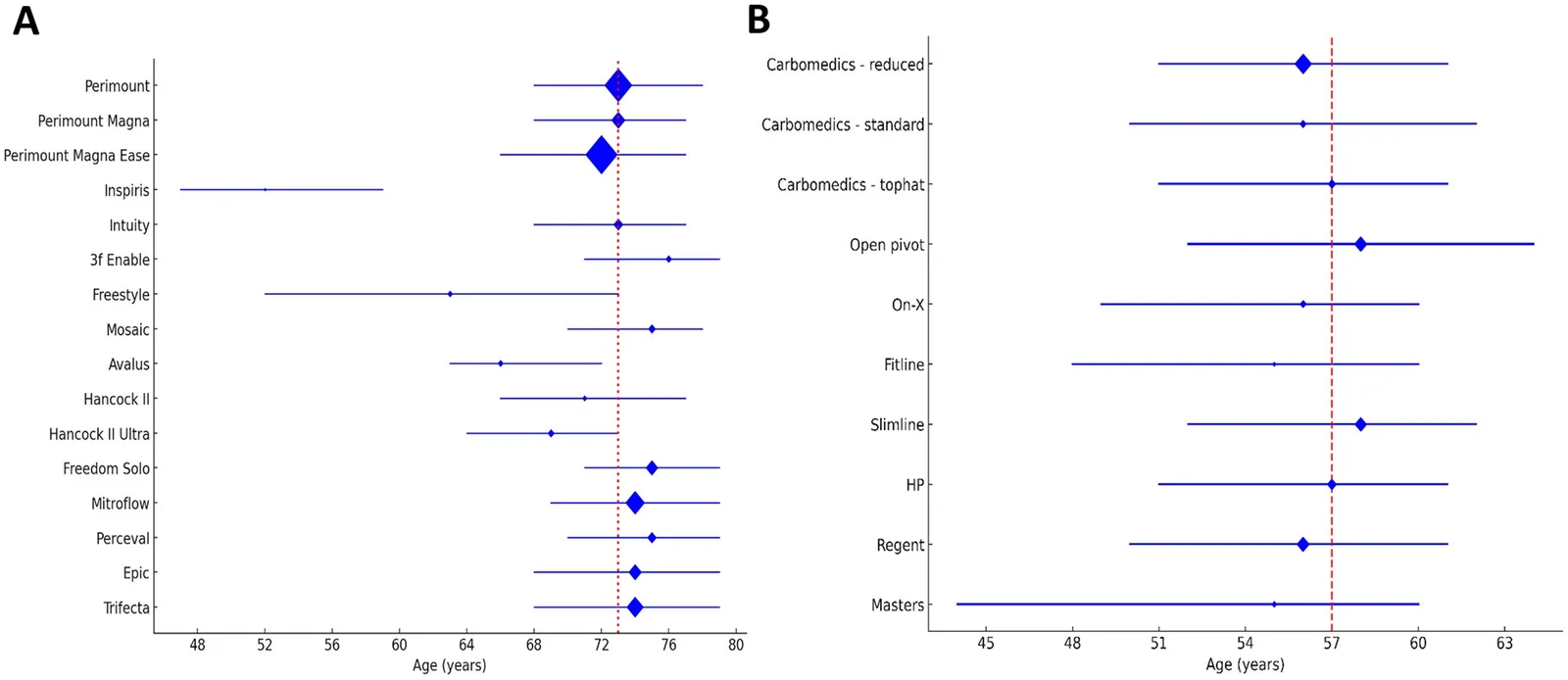
Median Age at Surgery. The size of the diamond is proportionate to the sample size. (A) By model of biological aortic valve prosthesis. (B) By model of mechanical aortic valve prosthesis

The median duration of follow-up for mortality was 71 months [IQR 52-105]. In total, 2707 deaths were included in the survival analysis. Adjusted 1-, 5-, and 10-year survival rates were 97.4% (CI 97.1%-97.7%), 89.2% (88.3%-90.2%), and 70.1% (67.8%-72.5%), respectively, with statistically significant differences in 10-year survival across BP models (**Figure 5A**; **Table 2**). Perimount Magna Ease showed the highest adjusted 10-year survival of 72.6% (70.1%-75.2%) after 10 years and was used as the reference. Both Intuity Elite (HR 0.89, CI 0.61-1.29, *P* > .05; 10-year survival not available (n.a.)) and Medical Epic (HR 0.97, CI 0.81-1.17, *P* > .05; 10-year survival 70.0%, CI 65.4%-74.9%) valves showed no statistical differences in survival compared to the Perimount Magna Ease. The following BP models showed lower survival rates compared to the reference: Perimount (HR 1.32, CI 1.19-1.48, *P* < 0.01; 10-year survival 61.4%, CI 58.8%-64.5%), Perimount Magna (HR 1.23, CI 1.06-1.43, *P* < 0.01; 10-year survival 65.1%, CI 58.5%-64.5%), Freedom Solo (HR 1.83, CI 1.57-2.14, *P* < 0.01; 10-year survival 51.5%, CI 46.0%-57.8%), Mitroflow (HR 1.83, CI 1.63-2.06, *P* < 0.01; 10-year survival 50.6%, CI 46.6%-55.3%), Perceval (HR 1.42, CI 1.06-1.90, *P* < 0.05; 10-year survival n.a.), and Trifecta (HR 1.27, CI 1.09-1.49, *P* < 0.01; 10-year survival 63.1%, CI 54.6%-73.1%).

**Table 2.** Adjusted Estimated Survival and Freedom From Reintervention at 1-, 5-, and 10-Years After Intervention Per Prosthesis Type

	Survival			Freedom from reintervention		
	1-year	5-year	10-year	1-year	5-year	10-year
**Biological prosthesis**	**97.4 (97.1-97.7)**	**89.2 (88.3-90.2)**	**70.1 (67.8-72.5)**	**99.9 (99.8-100)**	**99.7 (99.4-99.9)**	**99.2 (98.7-99.8)**
Perimount	97.1 (96.4-97.7)	86.6 (85.0-88.1)	61.4 (58.5-64.5)	99.6 (99.4-99.8)	99.2 (98.8-99.5)	98.2 (97.5-98.9)
Perimount Magna	96.4 (95.1-97.7)	88.8 (86.5-91.1)	65.1 (61.1-69.4)	99.8 (99.6-100)	99.5 (99.1-100)	99.3 (98.8-99.8)
Perimount Magna Ease	97.2 (96.7-97.7)	88.2 (87.1-89.4)	72.6 (70.1-75.2)	99.6 (99.4-99.8)	99.1 (98.8-99.4)	98.2 (97.6-98.8)
Intuity	97.6 (96.0-99.3)	88.2 (83.5-93.2)	n.a.	98.1 (96.9-99.4)	98.1 (96.9-99.4)	n.a.
Freedom Solo	95.2 (93.6-96.9)	81.6 (78.4-84.9)	51.5 (46.0-57.8)	99.0 (98.2-99.8)	98.1 (96.9-99.2)	93.2 (90.3-96.2)
Mitroflow	95.1 (94.0-96.3)	82.0 (79.8-84.3)	50.7 (46.6-55.3)	99.2 (98.7-99.6)	96.9 (95.8-97.9)	90.5 (87.9-93.3)
Perceval	96.1 (94.1-98.2)	85.5 (81.4-89.9)	n.a.	99.2 (98.3-100)	97.7 (95.0-100)	n.a.
Epic	97.8 (96.7-98.9)	91.2 (89.0-93.5)	70.0 (65.4-74.9)	99.3 (98.8-99.9)	97.7 (96.6-98.9)	97.3 (96.1-98.7)
Trifecta	96.4 (95.3-97.5)	85.4 (83.1-87.7)	63.1 (54.6-73.1)	99.4 (99.0-99.8)	97.5 (96.5-98.5)	96.8 (95.5-98.1)
**Mechanical prosthesis**	**99.3 (98.8-99.7)**	**96.5 (95.0-98.0)**	**90.5 (86.9-94.3)**	**99.3 (98.8-99.8)**	**98.1 (97.0-99.2)**	**97.3 (95.7-98.9)**
Carbomedics Reduced	99.1 (98.5-99.8)	93.9 (91.8-95.9)	87.0 (83.1-91.0)	99.3 (98.7-99.8)	98.2 (97.2-99.3)	97.1 (95.5-98.8)
Open pivot	99.4 (98.7-100)	98.4 (97.1-99.7)	89.2 (85.1-93.4)	99.5 (98.7-100)	97.8 (96.1-99.6)	96.1 (93.6-98.7)
Slimline	99.5 (98.9-100)	97.0 (95.4-98.6)	87.5 (80.2-95.3)	99.4 (98.8-100)	97.6 (96.0-99.3)	96.5 (94.3-98.8)
HP	99.0 (98.0-100)	94.0 (91.1-97.0)	87.3 (82.0-92.9)	97.3 (95.3-99.3)	95.4 (92.6-98.3)	94.9 (91.8-98.1)
Regent	98.7 (97.7-99.7)	96.4 (94.6-98.2)	91.2 (87.6-94.8)	99.0 (98.0-99.0)	96.7 (94.7-98.8)	95.9 (93.5-98.4)

Numbers shown are percentages with 95% confidence interval. Adjusted estimates were obtained from multivariable models that included age, EuroSCORE I, body surface area, and valve size as covariates.

Abbreviation: n.a., not available.

in bold mean values for all types of biological prosthesis and mechanical prosthesis combined

The median duration of follow-up for reintervention on the aortic valve was 66 months [IQR 37-99]. A total of 319 reinterventions were included in the analysis. Adjusted 1-, 5-, and 10-year freedom from reintervention rates, accounted for mortality as a competing risk, were 99.9% (CI 99.8%-100%), 99.7% (99.4%-99.9%), and 99.2% (98.7%-99.8%). The Perimount Magna valve showed the highest adjusted 10-year freedom from reintervention (10 y; 99.3%, CI 98.8%-99.8%) and was used as the reference. The freedom from reintervention of all other BP models differed significantly from the reference: Perimount (HR 2.38, CI 1.08-5.24, *P* < 0.05; 10 y 98.2%, CI 97.5%-98.9%), Perimount Magna Ease (HR 2.55, CI 1.18-5.52, *P* < 0.05; 10 y 98.2%, CI 97.6%-98.8%), Intuity Elite (HR 8.63, CI 3.16-23.56, *P* < 0.01, 10 y n.a.), Freedom Solo (HR 8.00, CI 3.55-18.07, *P* < 0.01; 10 y 93.2%, CI 90.3%-96.2%), Mitroflow (HR 11.1, CI 5.17-24.1, *P* < 0.01; 10 y 90.5%, CI 87.9%-93.3%), Perceval (HR 5.42, CI 1.70-17.2, *P* < 0.01; 10 y n.a.), Epic (HR 4.32, CI 1.86-10.2, *P* < 0.01, 10 y 97.3%, CI 96.1%-98.7%), and Trifecta (HR 6.01, CI 2.67-13.5, *P* < 0.01; 10 y 96.8%, CI 95.5%-98.1%) (**Figure 5A**).

### Mechanical prosthetic aortic valves

MP’s were implanted in 3601 patients during the study period. The Carbomedics Reduced was the most frequently implanted MP (24.0%; *n* = 866), followed by the Open Pivot (12.9%, *n* = 466), Regent (12.7%; *n* = 458), and Slimline (11.3%; *n* = 408) prostheses (**Table S2**). There were no differences in age at implantation or logistic EuroSCORE I across models (**Figures 2B**  **and**  **3B**). The distribution of MP size, ranging from 19 to 29 mm, is shown in **Figure 4**.

**Figure 3.**
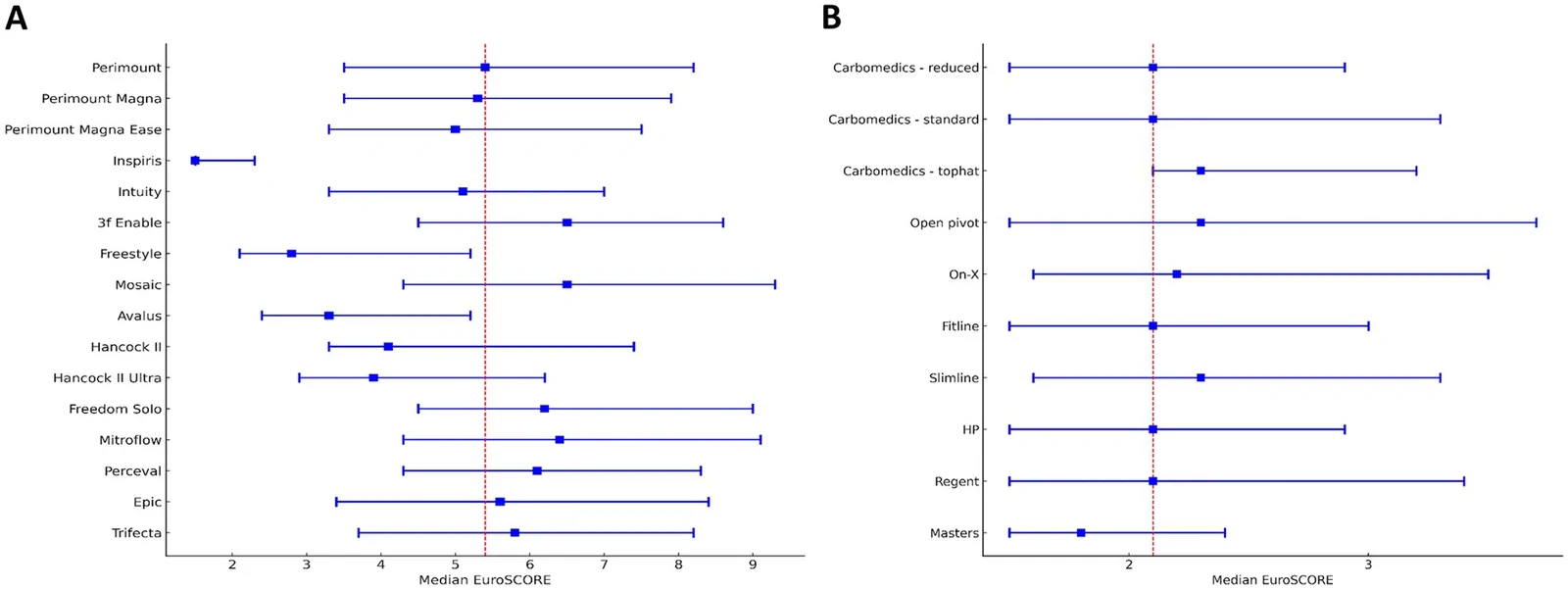
Median EuroSCORE-I. (A) By model of biological aortic valve prosthesis. (B) By model of mechanical aortic valve prosthesis

**Figure 4.**
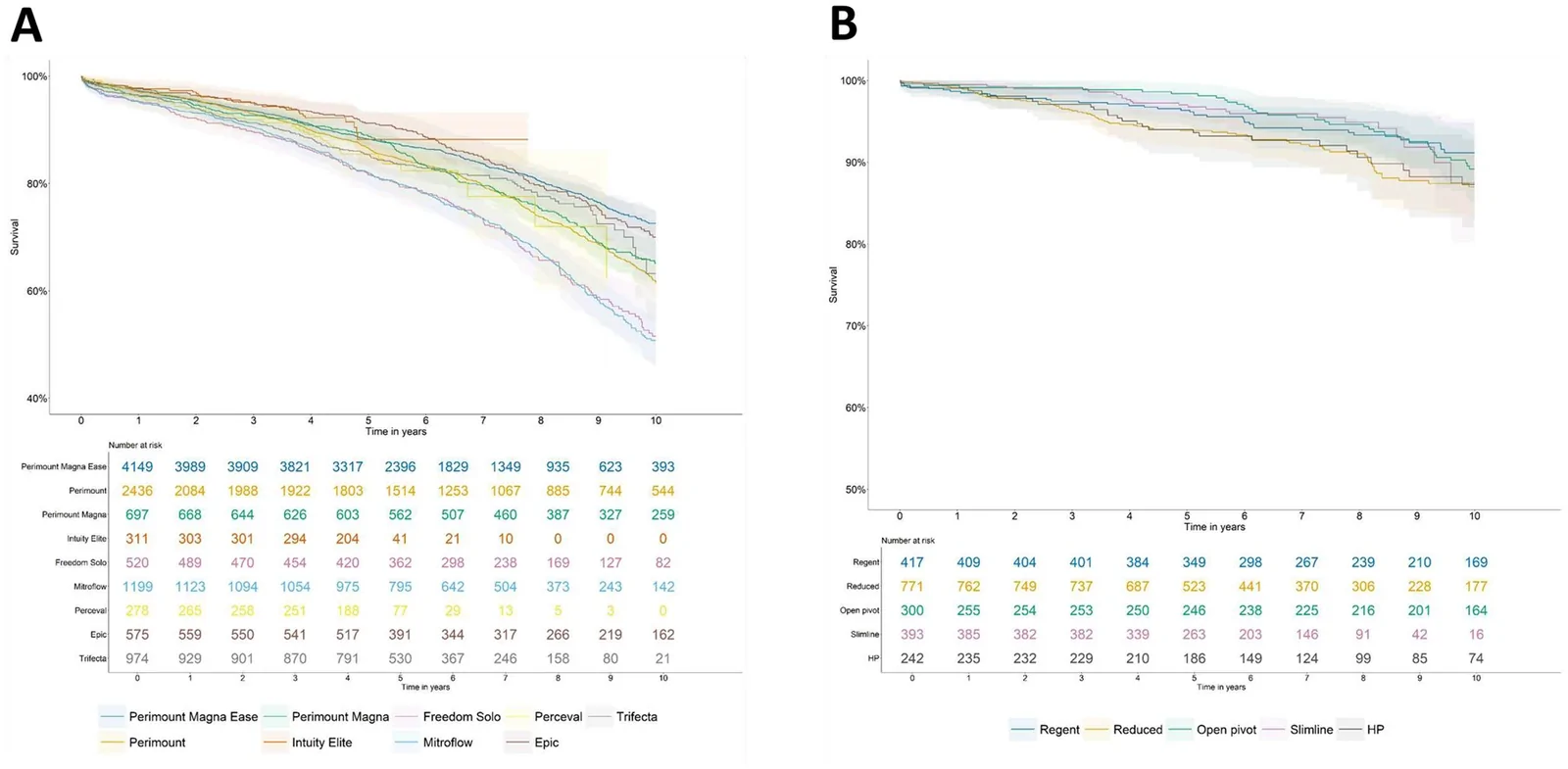
Survival Curves Showing Estimated Survival After Surgical Aortic Valve Replacement by Type of Valve, Adjusted for Age, Body Surface Area, Valve Size, and EuroSCORE I. (A) For biological aortic valve prostheses. (B) For mechanical aortic valve prostheses

The median duration of follow-up for mortality was 91 months [IQR 58-120] and 214 deaths were included in the survival analysis. Adjusted 1-, 5-, and 10-year survival rates were 99.3% (CI 98.8%-99.7%), 96.5% (95.0%-98.0%), and 90.5% (86.9%-94.3%), respectively (**Table 2**). Compared to the reference model of MP, which was the Regent (adjusted 10-year survival of 91.2%, CI 87.6%-94.8%), the majority of the other models did not show a statistically significant difference in survival: Open Pivot (HR 1.09, CI 0.67-1.76; 10-year survival 89.2%, CI 85.1%-93.4%), Slimline (HR 0.92, CI 0.56-1.52; 10-year survival 87.5%, CI 80.2%-95.3%), and HP (HR 1.47, CI 0.88-2.47, *P*-value >.05; 10-year survival 87.3%, CI 82.0%-92.9%) (**Figure 5B**). Only the Carbomedics Reduced showed a difference in survival (HR 1.55, CI 1.01-2.36, *P*-value <.05; 10-year survival 87.0%, CI 83.1%-91.0%) to the reference model of MP.

**Figure 5.**
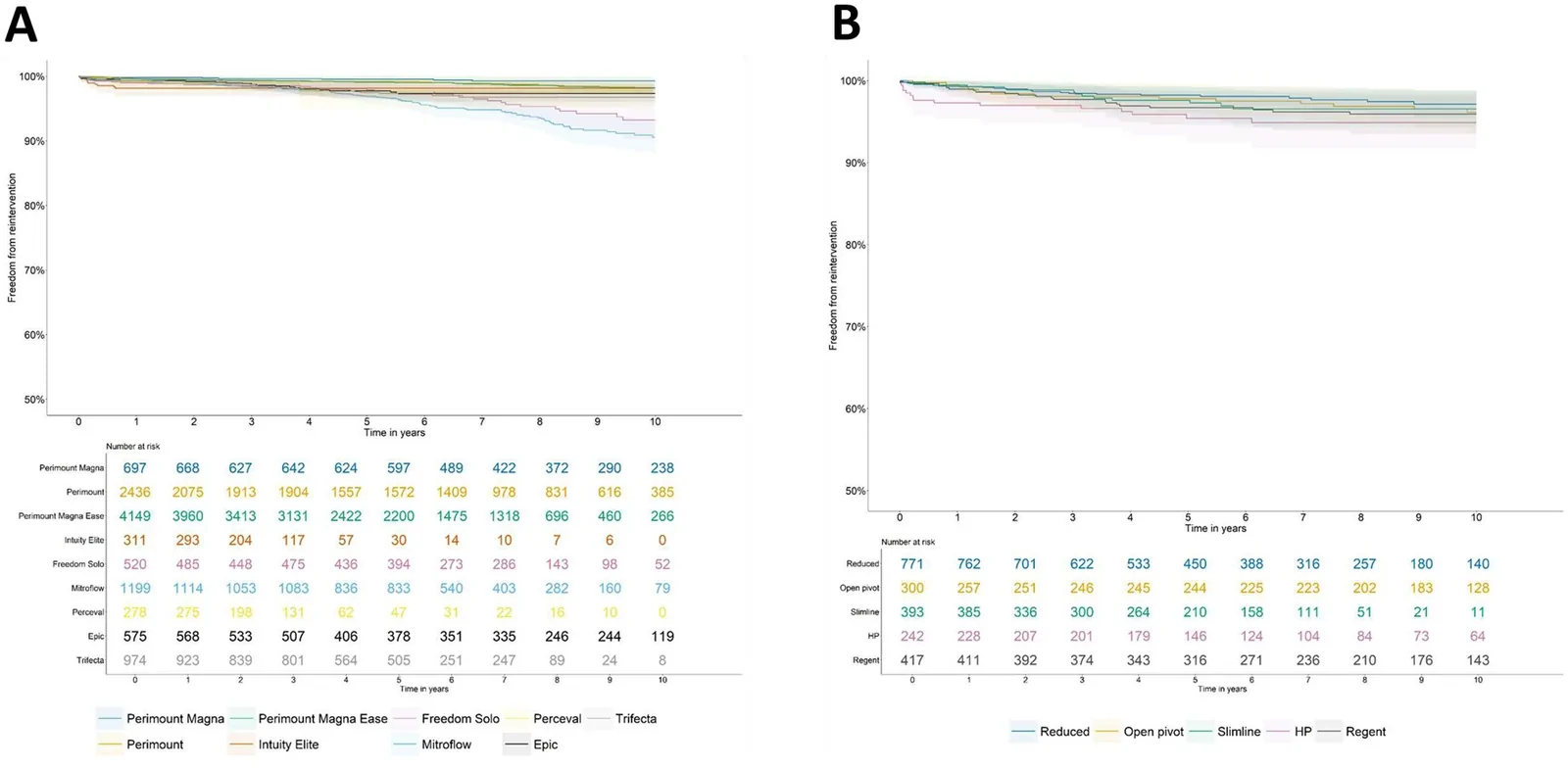
Survival Curves Showing Estimated Freedom from Reintervention After Surgical Aortic Valve Replacement by Type of Valve, Adjusted for Age, Body Surface Area, Valve Size, and EuroSCORE I, and Death Accounted for as a Competing Risk. (A) For biological aortic valve prostheses. (B) For mechanical aortic valve prostheses

The median duration of follow-up for reintervention on the aortic valve was 82 months [IQR 48-119], and a total of 86 reinterventions were included. The adjusted 1-, 5-, and 10-year freedom from reintervention rates were 99.3% (CI 98.8%-99.8%), 98.1% (97.0%-99.2%), and 97.3% (95.7%-98.9%), respectively. The MP with the highest adjusted 10-year freedom of reintervention (97.1%, CI 95.5%-98.8%) was the Carbomedics Reduced valve, and was used as the reference (**Figure S1**). Only the HP valve showed a significant difference in freedom from reintervention compared to the reference (HR 2.13, CI 1.11-4.10, *P* = .02; 10 y 94.9%, CI 91.8%-98.1%). All other valves showed similar rates of reintervention to the reference: Open Pivot (HR 1.36, CI 0.68-2.71; 10 y 96.1%, CI 93.6%-98.7%), Slimline (HR 1.36, CI 0.68-2.71; 10 y 96.5%, CI 94.3%-98.8%) and Regent (HR 1.57, CI 0.83-2.98; 10 y 95.9%, CI 93.5%-98.4%).

### Sensitivity analyses

Assessment of the proportional hazards assumption using Schoenfeld residuals demonstrated evidence of non-proportional hazards in all models. Covariates showed significant time-dependent effects and the global tests were significant for all models (all *P* < .05), indicating that HRs should be interpreted as average effects over the study period.

Inclusion of a proxy for PPM in the BP cohort did significantly improve the model fit for survival (*P* = .003) although no consistent association across PPM quartiles was observed. In the competing-risk analyses for reintervention, the lowest indexed valve size quartile was associated with a higher reintervention risk (HR 1.71), while the main effect estimates remained stable. In the MP cohort, inclusion of a proxy for PPM did not significantly improve model fit for survival and showed no consistent association with reintervention risk. Overall, the main effect estimates remained largely unchanged after adjustment for PPM.

## DISCUSSION

This study was performed to assess the long-term outcomes of different models of mechanical and biological prosthetic aortic valves and found important distinctions in survival and reintervention rates between different valve models. Concerning BP, in our study, the most favourable 10-year survival was found after SAVR with the Perimount Magna Ease and Epic valve prostheses (72.6% and 70.0%, respectively). Previous studies on the Perimount Magna Ease have reported 10-year survival rates within a similar range of 66.7% to 77.3%,^20–22^ although a much lower survival of 56.8% has also been reported.^23^ For the Epic prosthesis, Lehmann et al^24^ reported a 41.7% 10-year survival, considerably lower than the current study, but similar to the 44% 10-year survival with the Epic reported by Persson et al. Patient selection might play a role here. For example, the other 2 studies included “all comers” including emergent cases and patients with active endocarditis, while we opted to exclude cases with active endocarditis or concomitant bypass surgery.

The Perimount and Perimount Magna and Trifecta BP had similar outcomes in our study, with 10-year survival ranging between 61.4% and 65.1% and freedom from reintervention between 96.8% and 99.3%. This is considerably different from the previously reported 10-year survival of 52% for the Perimount prosthesis,^25^ and of 32% for the Trifecta prosthesis.^26^ We cannot definitely explain these inconsistencies, but patient selection is a likely cause, such as the fact that in both those studies, more than half of the patients underwent concomitant procedures, or were operated emergently. Possibly, device-specific failure mechanisms are more pronounced in different patient populations. The US registry-based study on the Trifecta reported a much lower freedom from valve reintervention of 82.4%, and in fact, the Trifecta prosthesis has been associated with an increased risk of reintervention.^27^ While in our study, the freedom from reintervention was 96.8%, which was significantly lower than the reference, but the difference was not as dramatic as in previous studies.

Although this study reports outcomes that are specific for the Netherlands, the results are quite consistent with the findings from registry-based studies from the United Kingdom and Sweden, suggesting at least reasonable external validity.^14–16^ In both the UK and the Swedish registries, the reported risk of death or reoperation was greater with the Mitroflow and reduced for the Perimount biological valve prostheses,^14^^,^^15^ while for MP, the UK registry did not find differences in long-term outcomes and the Swedish registry found a greater risk of death or reoperation with the Sorin Bicarbon group (mainly the Slimline prosthesis).^14^^,^^16^ These national registries, and the NHR, already offer high-volume, real-world, and rather complete data. The introduction of Unique Device Identification (UDI) codes will allow for more structural monitoring of the real-world performance of devices such as valve prostheses in real-world practice. Using data from nationwide quality registries in this perspective can be a valuable addition to current requirements for market access and mandatory post market surveillance as required in the Medical Device Regulation, as these registries include complete real-world populations and can facilitate long-term follow-up without additional registration burden. For example, at the time of writing of this study, the Mitroflow and Trifecta prostheses have been taken off the market due to significantly lower long-term survival and higher reintervention risks.^11^^,^^12^^,^^28–30^ The inferior performance of these prostheses was also found in the current study. “Real-time” analysis of the NHR database, or of other national registries, might help to identify inferior outcomes of valve prostheses sooner. Eventually, this may impact the choice of prosthetic aortic valve model in daily clinical practice.

Concerning MP, there was little variation in 10-year survival between different models in our study, ranging between 87.0% and 91.2%. Similarly, the variation in 10-year freedom from reintervention was small, ranging between 94.9% and 97.1%. Although some of these differences were statistically significant, the relatively low event rates (214 total deaths and only 86 reinterventions for all prosthetic valve models combined) reduce the reliability of these estimations, especially for freedom from reintervention. Hickey et al^14^ found no significant differences in outcomes between MP models. The finding from Granbom Koski et al,^16^ who found significantly worse outcomes with MP from the Bicarbon group (Sorin Slimline, Overline and Aortic LN MP), was not reproduced in the current study.

There are several important limitations of the current study that need mentioning. First of all, many models were not implanted frequently enough for reliable multivariable analysis, and even if only the more frequently implanted prosthetic valve models were selected, the number of events was quite low in the MP cohort. Unfortunately, information on the cause of death is unavailable in the NHR, while this could have provided valuable additional insights. Moreover, although registry data offer real-world outcomes, they are still observational data, so factors such as patient selection, surgeon or centre preference, as well as time-dependent effects on choice of prosthesis and on outcomes are potential sources of bias. Correction was performed for prosthesis size and BSA separately, not for a composite measure such as effective orifice area index, since this is considered an unreliable measure.^31^ Nevertheless, residual confounding by PPM cannot be fully excluded. Prosthetic aortic valve models may have the same size label but are still different in actual internal diameter; additionally, for the Perceval prosthesis the conversion to the more commonly used labels was a simplification. We have performed sensitivity analyses using a proxy for PPM, which did not alter the main results and conclusions of our study. In MP, no consistent association was observed, whereas in BP, the proxy showed an association with outcomes without affecting the overall estimates of the primary models. Residual confounding might also exist due to other factors that were not adjusted for, such as centre or surgeon effects, patient frailty or less invasive access methods, such as anterior thoracotomy or partial sternotomy, which may have influenced the outcomes of certain models used more frequently in those cases. We have adjusted our results as much as possible for confounding factors, but we acknowledge that adjusting for all the separate EuroSCORE-I variables would have been more accurate than adjusting only for the EuroSCORE-I itself. However, this information was missing for the earlier years of the study population. In addition, violation of the proportional hazards assumption was observed across multivariable models in our analyses, which is plausible given the long-term follow-up and the known time-dependent nature of valve degeneration. Therefore, the reported HRs should be interpreted as average effects over the follow-up period rather than as constant effects over time.

## CONCLUSION

In conclusion, significant differences in 10-year outcomes after SAVR were observed for the different prosthetic aortic valve models that were used in the NHR, especially for biological prosthetic aortic valve models, with survival varying from 50.7% to 72.6% and freedom from reintervention from 90.5% to 99.3%. These differences warrant further investigation, as they may impact the choice of biological aortic valve model in daily clinical practice. For different models of MP, there was no significant variation in outcomes.

## Supplementary Material

ivag240_Supplementary_Data

## Data Availability

The data underlying this article will be made available by the corresponding author upon reasonable request. Most of the data collected by the Netherlands Heart Registration is publicly available on www.hartenvaatcijfers.nl.
