# Supplementary material for: Ten-Year Reintervention-Free Survival After Surgical Aortic Valve Replacement for Different Prosthetic Aortic Valve Models: Comparison Using Real-World Data From the Netherlands Heart Registration
Source: Interdiscip Cardiovasc Thorac Surg. 2026 Aug 31;41(11):ivag240. doi: 10.1093/icvts/ivag240 (PMC13730593; doi:10.1093/icvts/ivag240)
Supplement: Supplementary file 1 [file ivag240_supplementary_data.zip › Supplementary Figure legend.docx]

*Supplementary Figure 1. Distribution of size (in mm) of implanted valve prosthesis*
