# Supplementary material for: Ten-Year Reintervention-Free Survival After Surgical Aortic Valve Replacement for Different Prosthetic Aortic Valve Models: Comparison Using Real-World Data From the Netherlands Heart Registration
Source: Interdiscip Cardiovasc Thorac Surg. 2026 Aug 31;41(11):ivag240. doi: 10.1093/icvts/ivag240 (PMC13730593; doi:10.1093/icvts/ivag240)
Supplement: Supplementary file 1 [file ivag240_supplementary_data.zip › Supplementary Table S1 (unchanged).docx]

Supplementary Table S1 Members of the Cardiothoracic Surgery Registration Committee of the Netherlands Heart Registration

| **Name** | **Center** |
| --- | --- |
| S. Bramer | Amphia Ziekenhuis |
| R.A.F. de Lind van Wijngaarden | Amsterdam UMC |
| B.M.J.A. Koene | Catharina Ziekenhuis |
| J.A. Bekkers | Erasmus MC |
| G.J.F. Hoohenkerk | HagaZiekenhuis |
| A.L.P. Markou | Isala |
| A. de Weger | Leids Universitair Medisch Centrum |
| P. Segers | Maastricht UMC+ |
| D. Stecher | Medisch Centrum Leeuwarden |
| R.G.H. Speekenbrink | Medisch Spectrum Twente |
| V.G. Hindori | Onze Lieve Vrouwe Gasthuis |
| W.W.L. Li | Radboudumc |
| E.J. Daeter | St. Antonius Ziekenhuis |
| M.M. Mokhles | UMC Utrecht |
| I.J. Wijdh – den Hamer | Universitair Medisch Centrum Groningen |
