# Supplementary material for: Ten-Year Reintervention-Free Survival After Surgical Aortic Valve Replacement for Different Prosthetic Aortic Valve Models: Comparison Using Real-World Data From the Netherlands Heart Registration
Source: Interdiscip Cardiovasc Thorac Surg. 2026 Aug 31;41(11):ivag240. doi: 10.1093/icvts/ivag240 (PMC13730593; doi:10.1093/icvts/ivag240)
Supplement: Supplementary file 1 [file ivag240_supplementary_data.zip › Supplementary Table S2 (new).docx]

Supplemental Table 2: Baseline characteristics of all implanted prosthetic aortic valves by model of biological and mechanical prostheses

|  |  |  | **Age** | **Sex - Male** | **BMI** | **BSA** | **EuroSCORE I** |
| --- | --- | --- | --- | --- | --- | --- | --- |
|  |  |  | **Median [Q1, Q3]** | **n (%)** | **Median [Q1, Q3]** | **Median [Q1, Q3]** | **Median [Q1, Q3]** |
| **Manufacturer** | **Biological prosthesis** | | | | | | |
| Edwards | Perimount | n=3102 | 73 [68, 78] | 1,847 (59.5%) | 27.2 [24.7, 30.0] | 1.94 [1.80, 2.09] | 5.45 [3.51, 8.22] |
| Edwards | Perimount Magna | n=713 | 73 [68, 77] | 360 (50.5%) | 27.1 [24.2, 30.1] | 1.90 [1.77, 2.06] | 5.33 [3.50, 7.87] |
| Edwards | Perimount Magna Ease | n=4,228 | 72 [66, 77] | 2,284 (54.0%) | 27.3 [24.7, 30.5] | 1.95 [1.82, 2.09] | 4.96 [3.25, 7.48] |
| Edwards | Inspiris | n=11 | 52 [48.5, 58] | 9 (81.8%) | 24.2 [22.3, 27.1] | 1.95 [1.84, 2.04] | 1.51 [1.51, 2.23] |
| Edwards | Intuity | n=316 | 73 [68, 77] | 150 (47.5%) | 27.1 [24.6, 29.9] | 1.92 [1.78, 2.06] | 5.10 [3.32, 6.98] |
| Medtronic | 3f Enable | n=109 | 76 [71, 79] | 50 (45.9%) | 27.5 [24.1, 30.8] | 1.91 [1.79, 2.03] | 6.54 [4.50, 8.64] |
| Medtronic | Freestyle | n=77 | 63 [52, 73] | 40 (51.9%) | 27.5 [26.3, 29.7] | 1.93 [1.83, 2.10] | 2.75 [2.08, 5.18] |
| Medtronic | Mosaic | n=172 | 75 [70, 78] | 101 (58.7%) | 27.8 [24.3, 31.3] | 1.96 [1.80, 2.07] | 6.48 [4.32, 9.21] |
| Medtronic | Avalus | n=87 | 66 [63, 71.5] | 65 (74.7%) | 26.7 [24.7, 29.4] | 1.98 [1.88, 2.13] | 3.31 [2.41, 5.06] |
| Medtronic | Hancock II | n=59 | 71 [66.5, 76.5] | 34 (57.6%) | 25.2 [24.4, 26.1] | 1.99 [1.86, 2.10] | 4.05 [3.36, 7.39] |
| Medtronic | Hancock II Ultra | n=133 | 69 [64, 73] | 97 (72.9%) | 26.1 [24.2, 28.4] | 1.98 [1.85, 2.14] | 3.89 [2.86, 6.16] |
| Sorin | Freedom Solo | n=552 | 75 [71, 79] | 211 (38.2%) | 27.5 [24.8, 31.0] | 1.89 [1.75, 2.02] | 6.24 [4.48, 8.96] |
| Sorin | Mitroflow | n=1,441 | 74 [69, 79] | 629 (43.7%) | 27.7 [25.0, 30.8] | 1.90 [1.76, 2.05] | 6.35 [4.31, 9.09] |
| Corcym | Perceval | n=285 | 75 [70, 79] | 121 (42.5%) | 27.8 [25.1, 31.8] | 1.94 [1.80, 2.08] | 6.15 [4.34, 8.28] |
| Sorin | Other | n=1 | 55 [55, 55] | 1 (100%) | 24.8 [24.8, 24.8] | 1.92 [1.92, 1.92] | 2.27 [2.27, 2.27] |
| Sorin | Unknown | n=340 | 69 [63, 76] | 177 (52.1%) | 28.2 [24.8, 30.8] | 1.92 [1.77, 2.06] | 4.38 [2.80, 7.17] |
| St Jude | Epic | n=623 | 74 [68, 78] | 362 (58.1%) | 26.3 [24.0, 29.3] | 1.94 [1.80, 2.07] | 5.57 [3.42, 8.37] |
| St Jude | Trifecta | n=1,106 | 74 [68, 79] | 454(41.0%) | 27.4 [24.6, 30.5] | 1.90 [1.75, 2.04] | 5.84 [3.71, 8.20] |
|  | **Overall** | **n=13,355** | **73 [67, 78]** | **6,992 (52.4%)** | **27.3 [24.6, 30.4]** | **1.93 [1.79, 2.07]** | **5.38 [3.48, 8.02]** |
| **Manufacturer** | **Mechanical prosthesis** | | | | | | |
| Corcym | Carbomedics - Reduced | n=866 | 56 [51, 61] | 611 (70.6%) | 27.2 [24.6, 30.9] | 2.04 [1.89, 2.17] | 2.08 [1.51, 2.93] |
| Corcym | Carbomedics -Standard | n=108 | 56 [50, 62] | 79 (73.1%) | 26.1 [24.2, 30.2] | 2.05 [1.93, 2.17] | 2.08 [1.51, 3.24] |
| Corcym | Carbomedics - Tophat | n=157 | 57 [51, 61] | 61 (38.9%) | 28.0 [25.0, 30.1] | 1.94 [1.80, 2.10] | 2.28 [2.08, 3.19] |
| Corcym | Unknown | n=148 | 59 [51.8, 63] | 99 (66.9%) |  | 1.96 [1.79, 2.10] | 2.22 [1.51, 3.11] |
| Medtronic | Open pivot | n=466 | 58 [52, 64] | 287 (61.6%) | 26.3 [24.0, 31.2] | 2.00 [1.85, 2.11] | 2.27 [1.51, 3.65] |
| Medtronic | Other | n=103 | 54 [48, 58] | 71 (68.9%) | 28.1 [24.6, 32.2] | 2.02 [1.90, 2.18] | 2.08 [1.51, 2.44] |
| Artivion | On-X | n=104 | 56 [49, 60] | 66 (63.5%) | 26.7 [24.2, 30.4] | 1.96 [1.84, 2.20] | 2.23 [1.57, 3.53] |
| Corcym | Fitline | n=27 | 55 [49, 59.5] | 21 (77.8%) | 26.0 [23.3, 27.2] | 2.08 [1.96, 2.16] | 2.10 [1.51, 2.90] |
| Corcym | Slimline | n=408 | 58 [52, 62] | 257 (63.0%) | 27.1 [24.3, 30.3] | 1.99 [1.82, 2.14] | 2.27 [1.63, 3.33] |
| Sorin | Other | n=213 | 59 [52, 64] | 133 (62.4%) | 25.8 [24.0, 30.9] | 1.97 [1.82, 2.14] | 2.27 [1.67, 3.65] |
| Sorin | Unknown | n=80 | 54 [47, 60] | 54 (67.5%) | 27.8 [24.6, 29.7] | 1.96 [1.83, 2.13] | 2.08 [1.51, 3.12] |
| St Jude | HP | n=242 | 57 [51, 61] | 178 (73.6%) | 26.9 [24.5, 30.7] | 2.02 [1.90, 2.14] | 2.08 [1.51, 2.85] |
| St Jude | Regent | n=458 | 56 [50, 61] | 261 (57.0%) | 27.2 [24.8, 31.1] | 1.98 [1.81, 2.13] | 2.08 [1.51, 3.37] |
| St Jude | Masters | n=59 | 55 [44, 59.5] | 44 (74.6%) | 28.1 [24.6, 31.4] | 2.04 [1.91, 2.13] | 1.83 [1.51, 2.38] |
| St Jude | Unknown | n=162 | 63 [57, 69] | 109 (67.3%) | 26.3 [24.9, 30.6] | 1.98 [1.82, 2.12] | 2.78 [1.91, 5.10] |
|  | **Overall** | **n=3,601** | **57 [51, 62]** | **2,331 (64.7%)** | **27.1 [24.4, 30.9]** | **2.00 [1.85, 2.14]** | **2.08 [1.51, 3.33]** |
