# Supplementary material for: Ten-Year Reintervention-Free Survival After Surgical Aortic Valve Replacement for Different Prosthetic Aortic Valve Models: Comparison Using Real-World Data From the Netherlands Heart Registration
Source: Interdiscip Cardiovasc Thorac Surg. 2026 Aug 31;41(11):ivag240. doi: 10.1093/icvts/ivag240 (PMC13730593; doi:10.1093/icvts/ivag240)
Supplement: Supplementary file 1 [file ivag240_supplementary_data.zip › Supplementary Table S3 (new).docx]

Supplementary Table S2 Details of multivariable models used to predict survival and freedom from reintervention

|  | Hazard Ratio | 95% confidence interval | | p-value |
| --- | --- | --- | --- | --- |
|  |  | Lower | Upper |  |
| **Biological prosthesis - Survival** | | | | |
| Concordance index: 0.65 |  |  |  |  |
| Age | 1.05 | 1.05 | 1.06 | <0.001 |
| BSA | 1.52 | 1.23 | 1.86 | <0.001 |
| EuroSCORE I | 1.04 | 1.03 | 1.05 | <0.001 |
| Valve size – 19 mm | 1.41 | 1.13 | 1.74 | <0.01 |
| Valve size – 21 mm | 0.99 | 0.90 | 1.10 | 0.89 |
| Valve size – 23 mm | - | - | - | - |
| Valve size - 25 mm | 1.03 | 0.93 | 1.14 | 0.62 |
| Valve size - 27 mm | 0.97 | 0.83 | 1.13 | 0.70 |
| Valve size - 29 mm | 1.27 | 0.91 | 1.78 | 0.17 |
| Valve type – Perimount | 1.33 | 1.19 | 1.48 | <0.001 |
| Valve type – Perimount Magna | 1.23 | 1.06 | 1.43 | <0.01 |
| Valve type – Perimount Magna Ease | - | - | - | - |
| Valve type – Intuity Elite | 0.89 | 0.61 | 1.29 | 0.51 |
| Valve type – Freedom Solo | 1.83 | 1.57 | 2.14 | <0.001 |
| Valve type – Mitroflow | 1.83 | 1.63 | 2.06 | <0.001 |
| Valve type – Perceval | 1.42 | 1.06 | 1.90 | <0.05 |
| Valve type – Epic | 0.97 | 0.81 | 1.17 | 0.73 |
| Valve type – Trifecta | 1.27 | 1.09 | 1.49 | <0.01 |
| **Biological prosthesis – Freedom from reintervention** | | | | |
| Concordance index: 0.72 |  |  |  |  |
| Age | 0.94 | 0.93 | 0.95 | <0.001 |
| BSA | 2.69 | 1.60 | 4.52 | <0.001 |
| EuroSCORE I | 0.97 | 0.93 | 1.02 | 0.22 |
| Valve size – 19 mm | 1.36 | 0.71 | 2.59 | 0.23 |
| Valve size – 21 mm | 1.07 | 0.79 | 1.46 | 0.65 |
| Valve size – 23 mm | - | - | - | - |
| Valve size - 25 mm | 0.87 | 0.65 | 1.16 | 0.33 |
| Valve size - 27 mm | 1.03 | 0.71 | 1.49 | 0.89 |
| Valve size - 29 mm | 1.08 | 0.49 | 2.39 | 0.85 |
| Valve type – Perimount | 2.38 | 1.08 | 5.24 | <0.05 |
| Valve type – Perimount Magna | - | - | - | - |
| Valve type – Perimount Magna Ease | 2.55 | 1.18 | 5.52 | <0.05 |
| Valve type – Intuity Elite | 8.63 | 3.16 | 23.56 | <0.001 |
| Valve type – Freedom Solo | 8.00 | 3.55 | 18.07 | <0.001 |
| Valve type – Mitroflow | 11.13 | 5.17 | 24.06 | <0.001 |
| Valve type – Perceval | 5.42 | 1.70 | 17.24 | <0.01 |
| Valve type – Epic | 4.32 | 1.86 | 10.20 | <0.001 |
| Valve type – Trifecta | 6.01 | 2.67 | 13.53 | <0.001 |
| **Mechanical prosthesis - Survival** | | | | |
| Concordance index: 0.72 |  |  |  |  |
| Age | 1.08 | 1.06 | 1.10 | <0.001 |
| BSA | 1.46 | 0.72 | 2.95 | 0.30 |
| EuroSCORE I | 1.04 | 1.02 | 1.07 | <0.001 |
| Valve size – 19 mm | 2.85 | 1.53 | 5.29 | <0.001 |
| Valve size – 21 mm | 0.93 | 0.61 | 1.43 | 0.75 |
| Valve size – 23 mm | - | - | - | - |
| Valve size - 25 mm | 0.84 | 0.58 | 1.20 | 0.33 |
| Valve size - 27 mm | 1.27 | 0.83 | 1.93 | 0.27 |
| Valve size - 29 mm | 0.96 | 0.38 | 2.44 | 0.94 |
| Valve type – Carbomedics Reduced | 1.55 | 1.01 | 2.36 | <0.05 |
| Valve type – Open pivot | 1.09 | 0.67 | 1.76 | 0.73 |
| Valve type – Slimline | 0.92 | 0.56 | 1.52 | 0.75 |
| Valve type – HP | 1.47 | 0.88 | 2.47 | 0.14 |
| Valve type – Regent | - | - | - | - |
| **Mechanical prosthesis – Freedom from reintervention** | | | | |
| Concordance index: 0.62 |  |  |  |  |
| Age | 0.99 | 0.96 | 1.02 | 0.41 |
| BSA | 0.76 | 0.25 | 2.31 | 0.62 |
| EuroSCORE I | 0.99 | 0.94 | 1.05 | 0.78 |
| Valve size – 19 mm | 1.67 | 0.56 | 5.00 | 0.35 |
| Valve size – 21 mm | 1.13 | 0.56 | 2.28 | 0.74 |
| Valve size – 23 mm | - | - | - | - |
| Valve size - 25 mm | 1.77 | 1.00 | 3.15 | 0.05 |
| Valve size - 27 mm | 1.48 | 0.71 | 3.09 | 0.30 |
| Valve size - 29 mm | 0.55 | 0.08 | 4.21 | 0.58 |
| Valve type – Carbomedics Reduced | - | - | - | - |
| Valve type – Open pivot | 1.36 | 0.68 | 2.71 | 0.39 |
| Valve type – Slimline | 1.36 | 0.68 | 2.71 | 0.38 |
| Valve type – HP | 2.13 | 1.11 | 4.10 | <0.05 |
| Valve type – Regent | 1.57 | 0.83 | 2.98 | 0.17 |
